# Metabolic syndrome: a population-based study of prevalence and risk factors

**DOI:** 10.1038/s41598-024-54367-4

**Published:** 2024-02-17

**Authors:** Zahra Jamali, Fatemeh Ayoobi, Zahra Jalali, Reza Bidaki, Mohammad Amin Lotfi, Ali Esmaeili-Nadimi, Parvin Khalili

**Affiliations:** 1 Non-Communicable Diseases Research Center, Rafsanjan University of Medical Sciences, Rafsanjan, Iran; 2 Clinical Research Development Unit (CRDU), Niknafs Hospital, Rafsanjan University of Medical Sciences, Rafsanjan, Iran; 3 Occupational Safety and Health Research Center, NICICO, World Safety Organization and Rafsanjan University of Medical Sciences, Rafsanjan, Iran; 4 Clinical Research Development Unit (CRDU), Moradi Hospital, Rafsanjan University of Medical Sciences, Rafsanjan, Iran; 5 Department of Clinical Biochemistry, School of Medicine, Rafsanjan University of Medical Sciences, Rafsanjan, Iran; 6 Department of Psychiatry, Fellowship of Neuropsychiatry, Research Center of Addiction and Behavioral Sciences, Shahid Sadoughi University of Medical Sciences, Yazd, Iran; 7 Clinical Research Development Unit, Ali-Ibn Abi-Talib Hospital (CRDU), Rafsanjan University of Medical Sciences, Rafsanjan, Iran; 8 Department of Internal Medicine, Ali-Ibn Abi-Talib Hospital, Rafsanjan University of Medical Sciences, Rafsanjan, Iran; 9 Social Determinants of Health Research Center, Rafsanjan University of Medical Sciences, Rafsanjan, Iran; 10 Department of Epidemiology, School of Public Health, Rafsanjan University of Medical Sciences, Rafsanjan, Iran

**Keywords:** Metabolic syndrome, Young adults, Cigarette smoking, Opium, Alcohol, Tobacco, Rafsanjan Youth Cohort Study, Prospective Epidemiological Research Studies in IrAN (PERSIAN), Health care, Medical research, Risk factors

## Abstract

The association between personal habits and metabolic syndrome (MetS) remains controversial. This study aimed to assess the prevalence of MetS among youths and its association with cigarette, tobacco, opium, and alcohol consumption in the Rafsanjan Youth Cohort Study (RYCS). The current cross-sectional study was based on data from RYCS, as part of the Rafsanjan Cohort Study (RCS). RCS is a branch of the prospective epidemiological research studies in Iran (PERSIAN). In the present study, 2843 youths aged 15–35 were included. MetS was diagnosed using the international diabetes federation (International IDF), National Cholesterol Education Panel- Adult Treatment Panel III (NCEP-ATPIII), and Iranian criteria (IDF Iranian). Binary logistic regression models were performed to estimate odds ratios (ORs) and confidence intervals (CIs). The prevalence of MetS was 7.67%, 7.14%, and 10.13% based on NCEP-ATPIII, IDF Iranian, and International IDF criteria respectively. The odds of MetS according to international IDF and Iranian IDF in the alcohol-drinking group in the last 12 months (OR: 1.51, 95%CI 1.02–2.21, OR: 1.66, 95%CI 1.11–2.48 respectively) were greater compared with the non-drinking group. The odds of having high TG in the alcohol-drinking group in the last 12 months was 1.53 times higher than the control group (OR = 1.53, 95% CI: 1.20–1.94). Furthermore, the odds of having high waist circumference (WC) according to IDF International was significantly higher in the tobacco-smoking group in the last 12 months and in the tobacco-smoking group in the last 12 months daily (OR: 1.23, 95%CI 1.01–1.49 and OR: 1.41, 95%CI 1.01–1.98 respectively) compared to the control groups. The prevalence of MetS was 7.67%, 7.14%, and 10.13% based on NCEP-ATPIII, IDF Iranian, and International IDF criteria respectively. The odds of MetS and high TG were greater in the alcohol-drinking group in the last 12 months compared with the non-drinking group. The odds of high WC in the last 12 months, were greater in the tobacco-smoking group compared with the non-smoking group. However, more longitudinal studies are needed to verify the associations observed in the current study.

## Introduction

Today, Metabolic syndrome (MetS) is determined as the greatest public health challenge in both developed and developing countries ^1^. MetS is a major risk factor for cardiovascular disease, stroke, and diabetes ^2,3^. Additionally, abdominal obesity, insulin resistance, hyperlipidemia, hyperglycemia, and hypertension as metabolic syndrome criteria, are defined as a set of cardiovascular risk factors. A person with MetS must have at least three of these factors: high waist, hypertriglyceridemia, decreased high-density lipoprotein (HDL (, increased blood pressure (BP), and hyperglycemia ^4^. There are several definitions of MetS by different organizations such as the International Federation for Diabetes (IDF) and National Cholesterol Education Panel-Adult Treatment Panel III (NCEP-ATP III) ^5–7^. According to the IDF, the global prevalence of MetS is about 25% based on race, age, and sex factors ^8^.

Although many treatment options have been considered for the management of MetS, lifestyle modification is the first line of treatment for this disorder ^9^. Alcohol drinking is one of the most prevalent lifestyle habits globally. Previous studies reported that alcohol consumption is a risk factor for MetS ^10^. However, the association between alcohol consumption and MetS is controversial ^10–12^. Also, several previous studies showed that cigarette and tobacco smoking induce insulin resistance ^13–15^ and increase the risk of abdominal obesity ^16,17^. On the other hand, there are many controversies about the effects of opium on blood lipids ^18,19^, blood pressure levels ^20,21^, and the risk of CVD ^22,23^. Opioids are the second most widely used substance in some Asian countries due to high accessibility and misconceptions about their beneficial effects on lipid factors, glucose, and blood pressure ^24,25^.

Epidemiological studies have reported the prevalence of MetS at about 5–7% in young people worldwide ^4^. The prevalence of this disorder has been estimated at 6.4–7.5% in Iranian young people ^26,27^. The presence of some MetS compounds at a young age can predict the development of this syndrome in the future ^28^. Therefore, estimating the prevalence of MetS in youths seems critical to reducing the risk of diseases, especially cardiovascular disease and its mortality ^4,8,24,26–28^. Previous studies have shown that sedimentary life and a high-fat diet increase the risk of MetS obviously, but the effects of alcohol, smoking, and opium on MetS are not inconclusive ^4,8,24,26–28^. Considering the inconsistent results, the effect of MetS disorder on the progression of cardiovascular disease, and limited related previous studies among youths ^4,8,24,26–28^, it is critical to determine the association between MetS and its risk factors. This study aimed to assess the prevalence of MetS among young people (by NCEP-ATPIII, IDF international, and IDF Iranian criteria) and its association with cigarette, tobacco, opium, and alcohol consumption in the Rafsanjan Youth Cohort Study (RYCS).

## Methods

### Subjects, study design, and ethical consideration

The current cross-sectional study was based on data from RYCS that encompassed 3006 young adults, aged 15–35 recruited in the Rafsanjan youth Cohort Study (RCS) ^29^, a constituent of the Prospective Epidemiological Research Studies in IrAN (PERSIAN) ^30^. The Persian Youth Cohort (PYC), which constitutes a subset of the PERSIAN study, includes 9000 young adults ^15–35^ from three cities in Iran. The study procedures, which comprised invitations, interviews, measurements, and physical examinations, were executed under the supervision of the Iranian Ministry of Health and Medical Education and the PERSIAN Cohort Central Committee. The present study was conducted in compliance with the PERSIAN cohort's protocol, and all the protocols of the present study were approved by the Ethics Committee of Rafsanjan University of Medical Sciences (IR.RUMS.REC.1400.101).

RYCS, as a part of PYC, is a prospective study and the participants are followed up for 10 years using phone and face-to-face interviews. This study aims to evaluate the incidence of psychiatric disorders such as suicide, depression, substance abuse, and injuries, and its associated factors. The baseline phase of the study was conducted from December 2016 to December 2018. The target population of the RYCS included 15–35-year-old individuals in both urban and rural areas of Rafsanjan who were randomly selected and invited to attend the study.

Participants entered the study voluntarily, and informed consent was obtained from every participant of RCS for the interview, physical examinations, bio-specimen collection, and the use of the collected data for research. For participants younger than 18, informed consent was acquired from the parents on their behalf, and all necessary measures were taken to ensure the confidentiality of the personal data of participants. In the present study, 2843 participants were included after excluding some subjects with missing information on MetS components and blood samples.

### Data collection and measurements

All study participants underwent comprehensive interviews, physical examinations, and biological sampling. The interviews were conducted using standardized, validated questionnaires by trained experts connected to a central server. The questionnaires covered topics such as demographic information, medical history, and habits such as smoking and substance use. Trained health professionals performed blood pressure and anthropometric measurements. Blood biochemical parameters were measured using a Biotechnia analyzer (BT 1500, Italy) at the Central Laboratory in Rafsanjan Cohort Center.

BMI was calculated as the ratio of weight (kg) to squared height (m^2^). Physical activity scores were evaluated based on weekly physical activity using a questionnaire derived from the short form of the International Physical Activity Questionnaire (IPAQ) ^31^.

In this study, opioids were defined as opium and opium derivatives such as heroin, sukhteh, and shireh. The Rafsanjan Cohort Study used detailed questionnaires to gather information on substance use and smoking status. The opioid derivatives reported among participants were heroin, opium (teriak), sukhteh, and shireh. Opium is prepared by air-drying the raw opium and consumed through pipe-smoking or orally. Sukhteh is a black dry residue attached to the teriak pipe post-smoking and is consumed orally. Shireh is a refined product of opium, which is a filtered product of boiling raw opium and sukhteh in water consumed via pipe smoking.

Personal habits including alcohol consumption, hookah smoking, and cigarette smoking were self-reported. The questionnaires about physical activity and personal habits used in this study were part of the PERSIAN cohort study questionnaires, which have been provided in the supplementary materials (supplementary document 1).

### Metabolic syndrome assessment

The prevalence of MetS was assessed in study participants using three distinct definitions, specifically those outlined by the NCEP-ATP III, IDF, and the IDF ethnic-specific cutoffs for the Iranian population. According to NCEP-ATP III, individuals with at least three of the following criteria are considered to have MetS: (1) a waist circumference of 102 cm or more in men and 88 cm or more in women, (2) triglyceride (TG) level of 150 mg/dl or higher or the use of medication, (3) HDL levels less than 40 mg/dl in men or 50 mg/dl in women or the use of medication, (4) hypertension (systolic blood pressure (BP) of 130 mmHg or more or diastolic BP of 85 mmHg or more) or the use of medication, and (5) hyperglycemia (fasting blood sugar levels of 100 mg/dl or more or the use of medication).

Under the IDF international definition, individuals with a waist circumference of 94 cm or more in men and 80 cm or more in women, plus at least two of the following criteria, were considered to have MetS: (1) TG level of 150 mg/dl or higher, (2) HDL levels less than 40 mg/dl in men or 50 mg/dl in women or the use of medication, (3) hypertension (systolic BP of 130 mmHg or more or diastolic BP of 85 mmHg or more) or the use of medication, and (4) hyperglycemia (fasting blood sugar levels of 100 mg/dl or more or the use of medication).

According to the IDF Iranian definition, individuals with a waist circumference of 95 cm or more in both genders, plus at least two of the following criteria, are considered to have MetS: (1) TG level of 150 mg/dl or higher, (2) HDL levels less than 40 mg/dl in men or 50 mg/dl in women or the use of medication, (3) hypertension (systolic BP of 130 mmHg or more or diastolic BP of 85 mmHg or more) or the use of medication, and (4) hyperglycemia (fasting blood sugar levels of 100 mg/dl or more or the use of medication) ^32^.

In the present study, among people without missing data of 5 metabolic syndrome indicators, people with 3 positive criteria out of 5 metabolic syndrome criteria were considered to have metabolic syndrome. It is worth noting that the frequency difference between the total number (total included subjects, n = 2843) and some of the covariates was related to the missing data.

### Statistical analysis

To describe the data, frequency (%) and mean (SD: standard deviation) were used for categorical variables and quantitative variables, respectively.

The individuals’ baseline characteristics were compared between the groups of our study (non-MetS and MetS) using a chi-square test (χ^2^) and a t-test for categorical and continuous variables, respectively. Cohen's kappa was measured to assess the level of concordance between MetS diagnostic criteria, including International IDF, Iranian IDF, and NCEP-ATP III. In addition, we used binary logistic analysis to determine the odds ratios (ORs) and the corresponding 95% confidence intervals (CI) for the association between MetS (with and without MetS) as response or dependent variable and personal habits as independent variables. Besides, we used bivariable (crude) and multivariable (adjusted) models in the regression analysis.

Potential confounding parameters were recognized based on subject matter knowledge and relevant epidemiological literature. Next, they were entered into the models sequentially according to their hypothesized strengths of association with personal habits and MetS. Variables with a p-value < 0.25 were considered confounders. The baseline (crude) model was stratified based on personal habits. The adjusted model was adjusted for confounding variables, including age (continuous variable), gender (male/female), education years (continuous variable), and physical activity score (continuous variable). Additionally, due to the very small amount of missing measured data to determine the metabolic syndrome (less than 5% of the data) and also for individual habits (less than 0.07%) and since the missing pattern was Missing At Random (MAR), multiple imputation strategy was used to analyze and estimate the missing data using the Markov Chain Monte Carl (MCMC) approach. All analyses were performed in Stata 14. All p-values were two-sided, with p-values < 0.05 and 95% confidence intervals considered statistically significant.

### Consent to participate

Written informed consent was obtained from the participants.

## Results

In the baseline phase of the Rafsanjan Youth Cohort study (RYCS), 3006 participants (1323 male and 1683 female) were included. Overall, laboratory measurements and other components related to metabolic syndrome of 2843 participants were collected. The participant's mean age was 25.82 ± 6.07. The prevalence of MetS was 7.67% (CI 95%:6.74–8.71), 7.14% (CI 95%:6.25–8.15), and 10.13% (CI 95%:9.07–11.30) based on NCEP-ATPIII, IDF Iranian and international IDF criteria respectively. The prevalence of the MetS according to NCEP-ATPIII and international IDF criteria was significantly higher in females than men. Overall, the kappa agreement coefficients between NCEP-ATPIII definition with Iranian IDF and international IDF were 75% and 80% respectively. Also, the kappa agreement coefficient between Iranian IDF and international IDF was 81% (Table 1).

**Table 1:** Prevalence of metabolic syndrome (MetS) and Kappa agreement coefficient by gender according to ATP III, IDF Iranian and IDF international criteria (n = 2843).

Criteria	Total (n = 2843)		Male (n = 1236)		Female (n = 1607)	
	MetS		MetS		MetS	
	Yes-n (%)	No -n (%)	Yes-n (%)	No -n (%)	Yes-n (%)	No -n (%)
NCEP- ATP III	1453 (90.42)	154 (9.58)	1172 (94.82)	64 (5.18)	2625 (92.33)	218 (7.67)
International IDF	1421 (88.43)	186 (11.57)	1134 (91.75)	102 (8.25)	2555 (89.87)	288 (10.13)
Iranian IDF	1501 (93.40)	106 (6.60)	1139 (92.15)	97 (7.85)	2640 (92.86)	203 (7.14)
	Kappa agreement coefficient					
Between NCEP-ATPIII and international IDF Criteria	80%		65%		88%	
Between Iranian IDF and international IDF Criteria	81%		97%		70%	
Between NCEP-ATPIII and Iranian Criteria	75%		68%		80%	

Table 2 shows the socio-demographic, personal habits, lifestyle, biochemical, and clinical characteristics in all the subjects with and without MetS according to all criteria. Prevalence of MetS was considerably more common in the older age groups and in people with a higher BMI. Opium consumption in lifetime was significantly higher among subjects with MetS according to Iranian IDF and international IDF criteria. The prevalence of MetS according to Iranian IDF was significantly higher among the cigarette-smoking group in the last 12 months, the cigarette-smoking group in the last 12 months daily and the cigarette-smoking group lifetime daily, and also among the alcohol-drinking group in the lifetime and in the alcohol-drinking group in the last 12 months.

**Table 2:** Demographic data and other life style related factors of the subjects with MetS according to of IDF international, IDF Iranian, and NCEP-ATP III criteria.

Variable	MetS (IDF international)			MetS (IDF Iranian)			MetS (NCEP-ATP III)		
	Yes (n %)	No (n %)	*p* value	Yes (n %)	No (n %)	*p* value	Yes (n %)	No (n %)	*p* value
Gender**—**no. (%)			**0.004**			0.199			** < 0.001**
Female	186 (64.58)	1421 (55.62)		106 (52.22)	1501 (56.86)		154 (70.64)	1453 (55.35)	
Male	102 (35.42)	1134 (44.38)		97 (47.78)	1139 (43.14)		64 (29.36)	1172 (44.65)	
Age groups—no. (%)			** < 0.001**			** < 0.001**			** < 0.001**
≤ 20	37 (12.85)	682 (26.69)		22 (10.84)	697 (26.40)		22 (10.09)	697 (26.55)	
21–30	124 (43.06)	1120 (43.84)		86 (42.36)	1158 (43.86)		96 (44.04)	1148 (43.73)	
≥ 30	127 (44.10)	753 (29.47)		95 (46.80)	785 (29.73)		100 (45.87)	780 (29.71)	
Mean ± SD	28.15 ± 5.24	25.55 ± 6.11	< 0.001**	28.55 ± 5.12	25.61 ± 6.09	< 0.001**	28.57 ± 4.96	25.59 ± 6.10	< 0.001**
Education years—no. (%)			**0.029**			**0.042**			0.134
≤ 12 years	195 (67.71)	1560 (61.10)		139 (68.47)	1616 (61.26)		145 (66.51)	1610 (61.38)	
≥ 13 years	93 (32.29)	993 (38.90)		64 (31.53)	1022 (38.74)		73 (33.49)	1013 (38.62)	
Physical activity—no. (%)			0.691			0.878			0.468
No	163 (56.60)	1405 (55.01)		110 (54.19)	1458 (55.25)		125 (57.34)	1443 (54.99)	
≤ Median*	67 (23.26)	578 (22.63)		49 (24.14)	596 (22.58)		52 (23.85)	593 (22.60)	
> Median	58 (20.14)	571 (22.36)		44 (21.67)	585 (22.17)		41 (18.81)	588 (22.41)	
BMI—no. (%)			** < 0.001**			** < 0.001**			** < 0.001**
< 25	42 (14.58)	1545 (60.47)		0 (0)	1587 (60.11)		19 (8.72)	1568 (59.73)	
25–29.9	119 (41.32)	691 (27.05)		84 (41.38)	726 (27.50)		83 (38.07)	727 (27.70)	
≥ 30	127 (44.10)	319 (12.49)		119 (58.62)	327 (12.39)		116 (53.21)	330 (12.57)	
Opium consumption in last 12 months—no. (%)			0.385			0.131			0.527
Yes	27 (9.38)	202 (7.91)		22 (10.84)	207 (7.84)		20 (9.17)	209 (7.96)	
No	261 (90.63)	2353 (92.09)		181 (89.16)	2433 (92.16)		198 (90.83)	2416 (92.04)	
Opium life time—no. (%)			**0.021**			**0.002**			0.177
Yes	53 (18.40)	343 (13.42)		43 (21.18)	353 (13.37)		37 (16.97)	359 (13.68)	
No	235 (81.60)	2212 (86.58)		160 (78.82)	2287 (86.63)		181 (83.03)	2266 (86.32)	
Opium consumption—no. (%)			0.373			0.094			0.819
Abuse	7 (2.43)	43 (1.69)		6 (2.96)	44 (1.67)		3 (1.38)	47 (1.79)	
Dependent	9 (3.13)	55 (2.16)		8 (3.94)	56 (2.12)		4 (1.83)	60 (2.29)	
No	272 (94.44)	2453 (96.16)		189 (93.10)	2536 (96.21)		211 (96.79)	2514 (95.92	
Cigarette smoking in last 12 months—no. (%)			0.878			**0.022**			0.905
Yes	52 (18.06)	452 (17.69)		48 (23.65)	456 (17.27)		38 (17.43)	466 (17.75)	
No	236 (81.94)	2103 (82.31)		155 (76.35)	2184 (82.73)		180 (82.57)	2159 (82.25)	
Cigarette smoking in last 12 months daily—no. (%)			0.245			**0.006**			0.642
Yes	18 (6.25)	120 (4.70)		18 (8.87)	120 (4.55)		12 (5.50)	126 (4.80)	
No	270 (293.75)	2435 (95.3)		185 (91.13)	2520 (95.45)		206 (94.50)	2499 (95.20)	
Cigarette smoking lifetime—no. (%)			0.558			0.135			0.212
Yes	79 (27.43)	743 (29.08)		68 (33.50)	754 (28.56)		55 (25.23)	767 (29.22)	
No	209 (72.57)	1812 (70.92)		135 (66.50)	1886 (71.44)		163 (74.77)	1858 (70.78)	
Cigarette smoking lifetime daily—no. (%)			0.249			**0.006**			0.855
Yes	25 (8.68)	175 (6.85)		24 (11.82)	176 (6.67)		16 (7.34)	184 (7.01)	
No	263 (91.32)	2380 (93.15)		179 (88.18)	2464 (93.33)		202 (92.66)	2441 (92.99)	
Alcohol drinking in last 12 months—no. (%)			0.570			**0.004**			0.288
Yes	46 (15.97)	376 (14.72)		44 (21.67)	378 (14.32)		27 (12.39)	395 (15.05)	
No	242 (84.03)	2179 (85.28)		159 (78.33)	2262 (85.68)		191 (87.61)	2230 (84.95)	
Alcohol drinking life time—no. (%)			0.990			**0.015**			**0.042**
Yes	69 (23.96)	613 (2399)		63 (31.03)	619 (23.45)		40 (18.35)	642 (24.46)	
No	219 (76.04)	1942 (76.01)		140 (68.97)	2021 (76.55)		178 (81.65)	1983 (75.54)	
Alcohol drinking—no. (%)			0.684			0.154			0.843
Abuse	15 (5.24)	109 (4.30)		14 (6.97)	110 (4.20)		9 (4.15)	115 (4.41)	
Dependent	7 (2.45)	75 (2.96)		7 (3.48)	75 (2.86)		5 (2.30)	77 (2.95)	
No	264 (92.31)	2353 (92.75)		180 (89.55)	2437 (92.94)		203 (93.550)	2414 (92.63)	
Tobacco in last 12 months—no. (%)			0.746			0.165			0.622
Yes	105 (36.46)	953 (37.43)		85 (41.87)	973 (36.98)		78 (35.78)	980 (37.46)	
No	183 (63.54)	1593 (62.57)		118 (58.13)	1658 (63.02)		140 (64.22)	1636 (62.54)	
Tobacco in last 12 months daily—no. (%)			0.897			0.114			0.889
Yes	21 (7.29)	181 (7.08)		20 (9.85)	182 (6.89)		16 (7.34)	186 (7.09)	
No	267 (92.71)	2374 (92.92)		183 (90.15)	2458 (93.11)		202 (92.66)	2439 (92.91)	
Tobacco lifetime—no. (%)			0.162			0.827			**0.015**
Yes	137 (47.57)	1326 (51.92)		106 (52.22)	1357 (51.42)		95 (43.58)	1368 (52.13)	
No	151 (52.43)	1228 (48.08)		97 (47.78)	1282 (48.58)		123 (56.42)	1256 (47.87)	
Tobacco lifetime daily—no. (%)			0.796			0.087			0.477
Yes	32 (11.11)	297 (11.62)		31 (15.27)	298 (11.29)		22 (10.09)	307 (11.70)	
No	256 (88.89)	2258 (88.38)		172 (84.73)	2342 (88.71)		196 (89.91)	2318 (88.30)	

Significant values are in [bold].

Chi-square test (χ^2^) was use for categorical variables (all variables except mean of age).

*Median of physical activity was 135 min/week.

**T-test was used for continues variables (mean of age).

Table 3 shows the association of opium usage, cigarette and tobacco smoking, and alcohol consumption with MetS according to all criteria, using the bivariate and multivariate logistic regression. In the crude regression model, the odds of MetS according to Iranian IDF in opium users in lifetime (OR: 1.74, 95%CI 1.22–2.48), alcohol drinkers in lifetime (OR:1.47, 95%CI 1.08–2.00), cigarette smoking in last 12 months, cigarette smoking in last 12 months daily and cigarette smoking in lifetime daily groups (OR:1.48, 95%CI 1.06–2.08), OR: 2.04, 95%CI 1.22–3.43, OR:1.89, 95%CI 1.19–2.95 respectively) were higher than the reference groups. These associations did not persist after adjustment for confounders (Table 3).

**Table 3:** Association of opium consumption, cigarette and tobacco smoking and alcohol drinking with Mets according to IDF international, IDF Iranian, and NCEP-ATP III criteria.

		MetS (IDF international)		MetS (IDF Iranian)		MetS (NCEP-ATP III)	
		Crude	Adjusted	Crude	Adjusted	Crude	Adjusted
Opium consumption in the last 12 months	Yes	1.21 (0.79–1.84)	0.98 (0.63–1.52)	1.43 (0.90–2.27)	0.92 (0.56–1.49)	1.17 (0.72–1.89)	1.01 (0.61–1.67)
	No	1	1	1	1	1	1
Opium in life time	Yes	1.45 (1.06–2.00)	1.20 (0.84–1.71)	1.74 (1.22–2.48)	1.06 (0.72–1.57)	1.29 (0.89–1.87)	1.15 (0.76–1.73)
	No	1	1	1	1	1	1
Opium abuse	Yes	1.47 (0.65–3.30)	1.18 (0.51–2.74)	1.83 (0.77–4.35)	0.96 (0.39–2.35)	0.76 (0.23–2.46)	0.67 (0.20–2.25)
	No	1	1	1	1	1	1
Opium dependent	Yes	1.48 (0.72–3.02)	1.27 (0.60–2.69)	1.92 (0.90–4.08)	1.08 (0.49–2.39)	0.79 (0.29–2.21)	0.75 (0.26–2.16)
	No	1	1	1	1	1	1
Cigarette smoking in the last 12 months	Yes	1.03 (0.75–1.41)	1.19 (0.83–1.70)	1.48 (1.06–2.08)	1.26 (0.85–1.85)	0.99 (0.68–1.41)	1.38 (0.90–2.10)
	No	1	1	1	1	1	1
Cigarette smoking in the last 12 months daily	Yes	1.35 (0.81–2.26)	1.39 (0.08–2.41)	2.04 (1.22–3.43)	1.45 (0.83–2.53)	1.16 (0.63–2.12)	1.41 (0.73–2.72)
	No	1	1	1	1	1	1
Cigarette smoking in lifetime	Yes	0.92 (0.70–1.21)	1.00 (0.72–1.39)	1.26 (0.93–1.71)	0.95 (0.66–1.38)	0.82 (0.60–1.12)	1.05 (0.72–1.55)
	No	1	1	1	1	1	1
Cigarette smoking in lifetime daily	Yes	1.29 (0.83–2.00)	1.25 (0.77–2.03)	1.89 (1.19–2.95)	1.23 (0.75–2.03)	1.05 (0.62–1.79)	1.19 (0.66–2.14)
	No	1	1	1	1	1	1
Alcohol drinking in the last 12 months	Yes	1.10 (0.79–1.54)	**1.51 (1.02–2.21)**	1.66 (1.17–2.35)	**1.66 (1.11–2.48)**	0.80 (0.53–1.21)	1.26 (0.78–2.03)
	No	1	1	1	1	1	1
Alcohol drinking lifetime	Yes	1.00 (0.75–1.33)	1.27 (0.87–1.84)	1.47 (1.08–2.00)	1.23 (0.82–1.83)	0.69 (0.49–0.99)	0.96 (0.61–1.52)
	No	1	1	1	1	1	1
Alcohol abuse	Yes	1.23 (0.70–2.14)	1.41 (0.78–2.55)	1.72 (0.97–3.07)	1.35 (0.73–2.49)	0.93 (0.47–1.86)	1.27 (0.62–2.73)
	No	1	1	1	1	1	1
Alcohol dependent	Yes	0.83 (0.38–1.82)	0.96 (0.42–2.17)	1.26 (0.57–2.78)	0.99 (0.44–2.26)	0.77 (0.31–1.93)	1.08 (0.42–2.82)
	No	1	1	1	1	1	1
Tobacco smoking in the last 12 months	Yes	0.96 (0.74–1.24)	1.14 (0.86–1.50)	1.23 (0.92–1.64)	1.16 (0.84–1.61)	0.93 (0.70–1.24)	1.27 (0.92–1.75)
	No	1	1	1	1	1	1
Tobacco smoking in the last 12 months daily	Yes	1.03 (0.65–1.65)	1.24 (0.76–2.03)	1.48 (0.91–2.40)	1.40 (0.84–2.33)	1.04 (0.61–1.77)	1.47 (0.84–2.57)
	No	1	1	1	1	1	1
Tobacco smoking in lifetime	Yes	0.84 (0.66–1.07)	0.95 (0.72–1.26)	1.04 (0.78–1.37)	0.87 (0.62–1.21)	0.71 (0.54–0.94)	0.90 (0.65–1.24)
	No	1	1	1	1	1	1
Tobacco smoking in lifetime daily	Yes	0.95 (0.65–1.40)	1.09 (0.72–1.67)	1.42 (0.95–2.12)	1.22 (0.79–1.88)	0.85 (0.54–1.34)	1.16 (0.70–1.91)
	No	1	1	1	1	1	1

Significant values are in [bold].

Adjusted model was adjusted for confounding variables age (continuous variable), gender (male/female), and education years (continuous variable).

MetS, metabolic syndrome.

In the adjusted regression model after adjustment for confounders, the odds of MetS according to international IDF in the alcohol-drinking group in the last 12 months (OR: 1.51, 95%CI 1.02–2.21) was greater compared with the non-drinking group. Also, the odds of MetS according to Iranian IDF after adjustment for confounders was almost twice greater in the alcohol-drinking group in the last 12 months (OR: 1.66, 95%CI 1.11–2.48) compared with the non-drinking group. No association was found in tobacco smoking with MetS according to all criteria, using the bivariate and multivariate logistic regression. We did not find any association between opium use, cigarette and tobacco smoking, alcohol consumption, and MetS according to NCEP-ATP III criteria (Table 3).

Additionally, we performed a sensitivity analysis based on age groups and included subjects over 18 years of age in the study, and based on this analysis, no difference was seen in the results of the age groups of 15–35 years and 19–35 years based on all three criteria, and the association between alcohol consumption and metabolic syndrome remained significant (Supplementary Table S1). Furthermore, we performed a sensitivity analysis based on missing data by multiple imputation strategy. However, the obtained estimates for missing variables were very similar to the complete case analysis (Supplementary Table S2).

Table 4 shows the results of crude and adjusted logistic regression analysis regarding the association between opium use, cigarette and tobacco smoking, and alcohol consumption with high TG, low HDL, high BP, and high FBS. The odds of having high TG in the alcohol-drinking group in the last 12 months was 1.53 times higher than the non-drinking group after adjusting for confounders (OR = 1.53, 95% CI: 1.20–1.94). In adjusted mode, the odds of having high TG in opium, cigarette, and tobacco smoking were not significant. Also, the association between opium, cigarette, and tobacco smoking and alcohol consumption with low HDL, high FBS, and high BP was not significant.

**Table 4:** Association of opium consumption, cigarette and tobacco smoking and alcohol drinking with high TG, high LDL, high BP and high FBS according to of IDF international, IDF Iranian, and NCEP-ATP III criteria.

		High TG		Low HDL		High BP		High FBS	
		Crude	Adjusted	Crude	Adjusted	Crude	Adjusted	Crude	Adjusted
Opium consumption in the last 12 months	Yes	**1.62 (1.22–2.15)**	0.97 (0.71–1.31)	**0.53 (0.32–0.87)**	0.88 (0.51–1.50)	0.96 (0.48–1.92)	0.66 (0.32–1.34)	0.170 (1.27–2.28)	1.23 (0.90–1.67)
	No	1	1	1	1	1	1	1	1
Opium in life time	Yes	**1.91 (1.53–2.39)**	1.04 (0.81–1.34)	**0.50 (0.34–0.74)**	0.98 (0.64–1.51)	0.91 (0.53–1.59)	0.55 (0.31–1.00)	**1.48 (1.17–1.88)**	0.98 (0.76–1.27)
	No	1	1	1	1	1	1	1	1
Opium abuse	Yes	**2.11 (1.20–3.73)**	0.94 (0.52–1.69)	**0.27 (0.07–1.13)**	0.98 (0.23–4.23)	1.50 (0.46–4.90)	0.85 (0.25–2.89)	1.59 (0.87–2.90)	0.91 (0.49–1.70)
	No	1	1	1	1	1	1	1	1
Opium dependent	Yes	**3.52 (2.13–5.80)**	1.66 (0.99–2.80)	**0.21 (0.05–0.87)**	0.87 (0.18–3.32)	0.76 (0.18–3.15)	0.45 (0.11–1.92)	1.12 (0.63–1.99)	0.68 (0.37–1.22)
	No	1	1	1	1	1	1	1	1
Cigarette smoking in the last 12 months	Yes	**1.99 (1.63–2.44)**	1.18 (0.94–1.48)	**0.40 (0.27–0.58)**	1.09 (0.71–1.67)	0.85 (0.51–1.41)	0.64 (0.37–1.11)	**1.29 (1.04–1.61)**	1.00 (0.79–1.28)
	No	1	1	1	1	1	1	1	1
Cigarette smoking in the last 12 months daily	Yes	**2.03 (1.43–2.88)**	1.02 (0.70–1.47)	**0.30 (0.13–0.68)**	1.20 (0.50–2.88)	0.88 (0.35–2.19)	0.60 (0.23–1.50)	1.14 (0.77–1.69)	0.76 (0.50–1.15)
	No	1	1	1	1	1	1	1	1
Cigarette smoking in lifetime	Yes	**1.96 (1.64–2.34)**	1.10 (0.89–1.36)	**0.40 (0.30–0.54)**	1.01 (0.72–1.42)	1.16 (0.78–1.73)	0.87 (0.54–1.39)	1.20 (0.99–1.45)	0.87 (0.69–1.08)
	No	1	1	1	1	1	1	1	1
Cigarette smoking in lifetime	Yes	**2.19 (1.63–2.93)**	1.05 (0.76–1.44)	**0.34 (0.18–0.65)**	1.35 (0.67–2.75)	0.71 (0.31–1.64)	0.44 (0.19–1.05)	1.25 (0.91–1.74)	0.80 (0.56–1.13)
	No	1	1	1	1	1	1	1	1
Alcohol drinking in the last 12 months	Yes	**2.34 (1.89–2.90)**	**1.53 (1.20–1.94)**	**0.27 (0.16–0.44)**	0.85 (0.50–1.46)	1.13 (0.68–1.87)	0.99 (0.57–1.71)	1.12 (0.88–1.43)	0.91 (0.70–1.19)
	No	1	1	1	1	1	1	1	1
Alcohol drinking in lifetime	Yes	**2.07 (1.72–2.48)**	1.08 (0.86–1.36)	**0.27 (0.19–0.40)**	0.99 (0.63–1.57)	1.27 (0.84–1.92)	0.98 (0.59–1.63)	**1.34 (1.10–1.63)**	0.97 (0.76–1.24)
	No	1	1	1	1	1	1	1	1
Alcohol abuse	Yes	**2.49 (1.73–3.58)**	1.41 (0.96–2.07)	**0.21 (0.08–0.58)**	0.84 (0.30–2.40)	1.70 (0.81–3.58)	1.37 (0.62–2.99)	1.32 (0.88–1.97)	0.65 (0.62–1.45)
	No	1	1	1	1	1	1	1	1
Alcohol dependent	Yes	**2.36 (1.51–3.69)**	1.38 (0.87–2.20)	**0.08 (0.01–0.57)**	0.31 (0.04–2.24)	1.60 (0.63–4.04)	1.31 (0.50–3.42)	0.81 (0.47–1.41)	0.59 (0.33–1.05)
	No	1	1	1	1	1	1	1	1
Tobacco smoking in the last 12 months	Yes	**1.53 (1.29–1.81)**	1.07 (0.88–1.30)	**0.62 (0.49–0.79)**	1.16 (0.89–1.52)	1.19 (0.82–1.74)	1.10 (0.73–1.67)	1.13 (0.94–1.35)	0.98 (0.81–1.20)
	No	1	1	1	1	1	1	1	1
Tobacco smoking in the last 12 months daily	Yes	**1.71 (1.27–2.30)**	1.18 (0.86–1.61)	**0.49 (0.28–0.85)**	1.26 (0.70–2.30)	1.39 (0.73–2.63)	1.28 (0.66–2.49)	1.10 (0.79–1.54)	0.95 (0.66–1.34)
	No	1	1	1	1	1	1	1	1
Tobacco smoking in lifetime	Yes	**1.57 (1.32–1.85)**	0.98 (0.80–1.19)	**0.56 (0.45–0.71)**	1.08 (0.85–1.38)	1.17 (0.80–1.70)	1.01 (0.66–1.55)	1.03 (0.86–1.22)	0.81 (0.66–1.00)
	No	1	1	1	1	1	1	1	1
Tobacco smoking lifetime daily	Yes	**1.95 (1.54–2.48)**	1.20 (0.93–1.55)	**0.39 (0.24–0.64)**	1.16 (0.69–1.97)	1.23 (0.72–2.12)	1.03 (0.58–1.84)	1.10 (0.84–1.44)	0.86 (0.64–1.14)
	No	1	1	1	1	1	1	1	1

Significant values are in [bold].

Adjusted model was adjusted for confounding variables age (continuous variable), gender (male/female), and education years (continuous variable).

MetS, metabolic syndrome; TG, triglyceride; HDL, high-density lipoprotein; FBS, fasting blood sugar; BP, blood pressure.

Table 5 shows the results of crude and adjusted logistic regression analysis regarding the association between opium use, cigarette and tobacco smoking, and alcohol consumption with high waist circumference based on all three criteria. The odds of having high waist circumference (WC) according to IDF international was significantly higher in tobacco smoking in the last 12 months and tobacco smoking in the last 12 months daily (OR: 1.23, 95%CI 1.01–1.49 and OR: 1.41,95%CI 1.01–1.98 respectively). The odds of having high WC according to IDF Iranian was significantly lower in tobacco smoking in the lifetime group (OR: 0.81, 95%CI 0.67–0.98).

**Table 5:** Association of opium usage, cigarette and tobacco smoking and alcohol drinking with high waist circumference (WC) according to of IDF international, IDF Iranian, and NCEP-ATP III criteria.

		High WC (IDF international)		High WC (IDF Iranian)		High WC (NCEP-ATP III)	
		Crude	Adjusted	Crude	Adjusted	Crude	Adjusted
Opium consumption in the last 12 months	Yes	0.96 (0.73–1.26)	1.15 (0.84–1.57)	1.33 (0.99–1.77)	0.90 (0.66–1.23)	0.74 (0.55–1.01)	0.83 (0.59–1.17)
	No	1	1	1	1	1	1
Opium in life time	Yes	0.83 (0.67–1.03)	1.04 (0.80–1.35)	**1.35 (1.07–1.70)**	0.85 (0.66–1.09)	**0.73 (0.57–0.93)**	0.88 (0.67–1.17)
	No	1	1	1	1	1	1
Opium abuse	Yes	**0.51 (0.29–0.91)**	0.77 (0.42–1.42)	1.45 (0.80–2.62)	0.82 (0.44–1.51)	**0.47 (0.23–0.97)**	0.75 (0.35–1.59)
	No	1	1	1	1	1	1
Opium dependent	Yes	**0.47 (0.28–0.79)**	0.77 (0.44–1.34)	1.37 (0.81–2.33)	0.83 (0.48–1.45)	**0.30 (0.14–0.64)**	0.52 (0.24–1.12)
	No	1	1	1	1	1	1
Cigarette smoking in the last 12 months	Yes	**0.46 (0.38–0.56)**	0.98 (0.77–1.24)	1.23 (0.99–1.52)	1.05 (0.83–1.34)	**0.49 (0.38–0.61)**	1.05 (0.80–1.39)
	No	1	1	1	1	1	1
Cigarette smoking in the last 12 months daily	Yes	**0.44 (0.31–0.63)**	0.88 (0.59–1.30)	1.41 (0.98–2.03)	1.03 (0.70–1.53)	**0.57 (0.38–0.87)**	1.29 (0.82–2.04)
	No	1	1	1	1	1	1
Cigarette smoking in lifetime	Yes	**0.46 (0.39–0.54)**	0.97 (0.78–1.20)	1.13 (0.94–1.35)	0.89 (0.71–1.11)	**0.45 (0.37–0.54)**	0.89 (0.70–1.14)
	No	1	1	1	1	1	1
Cigarette smoking in lifetime daily	Yes	**0.43 (0.32–0.58)**	0.77 (0.55–1.08)	1.26 (0.93–1.73)	0.85 (0.60–1.19)	**0.48 (0.33–0.69)**	0.96 (0.64–1.45)
	No	1	1	1	1	1	1
Alcohol drinking in the last 12 months	Yes	**0.42 (0.34–0.52)**	1.23 (0.95–1.59)	1.13 (0.90–1.43)	1.09 (0.84–1.42)	**0.35 (0.27–0.46)**	0.89 (0.66–1.22)
	No	1	1	1	1	1	1
Alcohol drinking in lifetime	Yes	**0.35 (0.29–0.42)**	0.91 (0.72–1.16)	1.07 (0.88–1.30)	0.83 (0.65–1.06)	**0.32 (0.25–0.40)**	0.77 (0.58–1.03)
	No	1	1	1	1	1	1
Alcohol abuse	Yes	**0.34 (0.23–0.50)**	0.85 (0.56–1.30)	1.06 (0.71–1.58)	0.85 (0.56–1.31)	**0.28 (0.16–0.49)**	0.73 (0.41–1.29)
	No	1	1	1	1	1	1
Alcohol dependent	Yes	**0.37 (0.23–0.59)**	0.97 (0.58–1.61)	1.23 (0.76–1.98)	1.03 (0.62–1.70)	**0.42 (0.24–0.76)**	1.11 (0.60–2.06)
	No	1	1	1	1	1	1
Tobacco smoking in the last 12 months	Yes	**0.58 (0.50–0.67)**	**1.23 (1.01–1.49)**	1.09 (0.92–1.30)	1.06 (0.88–1.29)	**0.56 (0.48–0.67)**	1.05 (0.86–1.29)
	No	1	1	1	1	1	1
Tobacco smoking in the last 12 months daily	Yes	**0.56 (0.42–0.76)**	**1.41 (1.01–1.98)**	1.31 (0.96–1.78)	1.29 (0.93–1.79)	**0.56 (0.39–0.79)**	1.25 (0.84–1.85)
	No	1	1	1	1	1	1
Tobacco smoking lifetime	Yes	**0.49 (0.42–0.57)**	0.99 (0.82–1.21)	0.94 (0.79–1.11)	**0.81 (0.67–0.98)**	**0.47 (0.40–0.55)**	0.83 (0.68–1.00)
	No	1	1	1	1	1	1
Tobacco smoking lifetime daily	Yes	**0.47 (0.37–0.60)**	1.14 (0.86–1.50)	1.22 (0.95–1.57)	1.09 (0.83–1.43)	**0.46 (0.35–0.62)**	1.07 (0.76–1.49)
	No	1	1	1	1	1	1

Significant values are in [bold].

Adjusted model was adjusted for confounding variables age (continuous variable), gender (male/female), and education years (continuous variable).

MetS, metabolic syndrome; WC, waist circumference.

## Discussion

Given the increasing prevalence of obesity in Iran, this study aimed to assess the prevalence of metabolic syndrome and its related risk factors among youths in a region in the southeast of Iran, based on NCEP-ATPIII, IDF Iranian, and international IDF criteria. According to our knowledge, this is the first study reporting the prevalence of MetS in Iranian youths in a large population-based study using three different criteria. Results of the present study showed that the prevalence of MetS in the Rafsanjan youth cohort population varied between 7.14% and 10.13% according to different criteria. The prevalence of MetS in the total population was 7.67%, 7.14%, and 10.13% based on NCEP-ATPIII, IDF Iranian, and International IDF criteria respectively. The highest prevalence was related to International IDF criteria and the lowest one was estimated by the Iranian IDF. The Kappa coefficient showed that there is good agreement between the three criteria. The main problem in the diagnosis of MetS is the different definitions of MetS and unavailability of universally accepted definition of this phenomenon ^33^. Therefore, MetS prevalence estimates may vary considerably between populations, due to their characteristics and different diagnostic criteria ^34^. Other studies evaluating definitions of MetS in youth reported variations from 2.7 to 3.8% ^35^ and from 0.3 to 26.4% ^36^. Melo et al., in 2023 showed that different criteria provide different estimates for the prevalence of MetS in adolescents and youth, reflecting the importance of establishing a consensus ^37^. Findings by Nolan’s study (a pooled analysis) highlighted that MetS was prevalent in young adults (18–30 years old) at about 4.8% (NCEP-ATPIII) and 7.0% (International IDF) ^4^. This difference between studies can be due to the different age ranges.

In our study, the prevalence of MetS in young females was higher than in young males. A meta-analysis of 69 studies in Iran presented the prevalence of MetS was higher in women compared to men ^38^.

The present study provides important data about the association between MetS and personal habits (cigarette, tobacco, opium, and alcohol consumption) in young adults. The findings indicated that, after adjustment for confounding variables, tobacco smoking in the last 12 months and also, in the last 12 months daily were associated with an increase in the odds of high WC (International IDF). Also, after adjustment for confounding variables, the odds of MetS were greater in the alcohol-drinking group in the last 12 months compared with the non-drinking group (International and Iranian IDF), and the odds of having high TG in the alcohol-drinking group in the last 12 months was higher than the non-drinking group.

Although in the univariable models, we observed a positive relationship between lifetime opium use and MetS based on the international and Iranian criteria, as well as between smoking and MetS based on the Iranian criteria, this association was not significant after controlling the effect of the confounders of age, gender, and education. Because these variables are correlated with exposures of interest in this study and are risk factors for MetS, for example, there is a correlation between education level with opium and cigarette consumption. So that people with lower education may be more opium and cigarette users, on the other hand, education level can be a risk factor for MetS. Because people with a lower level of education may have a lower level of information and a lower socio-economic status than people with a higher education, as a result, these people are more exposed to MetS, due to unhealthy diets and other unhealthy lifestyle behaviors. Therefore, it is important to consider the confounding effect of variables such as education when investigating the association between individual habits such as smoking and opium consumption with MetS.

Kan Sun et al. in a meta-analysis of prospective studies reported that heavy alcohol consumption was associated with an increased risk of MetS while very light alcohol consumption was associated with a reduced risk of MetS ^11^. In another study, results showed that the prevalence of MetS was high in participants with a median age of 47 years old who started drinking alcohol at 16 years old ^10^. Similarly, the present study showed the odds of MetS was almost twice greater in the alcohol-drinking group in the last 12 months compared with the non-drinking group.

The association between alcohol consumption and MetS according to the NCEP-ATPIII guidelines among 10,037 subjects was analyzed in a community-based Cohort of Korean adults. The very light alcohol-drinking group was significantly associated with a lower prevalence of MetS compared to the non-drinking group. Heavy alcohol drinking did not show a significant association with the prevalence of MetS but contributed to increased hyperglycemia and decreased low HDL cholesterol ^12^. Contrary to the results of the previous study, our results did not show a significant association between alcohol consumption and glucose and HDL cholesterol, but there was a positive association with MetS. The different results among previous studies can be due to differences in the diet, daily life stress, study design (cross-sectional or longitudinal), sample sizes, analytic methods, population characteristics, race, geographic area, personal habits measurement, diagnostic criteria for MetS, and different adjusting confounders.

One study in 2022 indicated that the prevalence of hypertriglyceridemia (33.2%), hyperglycemia (27.1%), and low HDL cholesterol (20.1%) was higher in alcohol users and excessive alcohol consumption had been associated with MetS and their components such as hypertension, dyslipidemia, diabetes or obesity ^10^. Similarly, the present study showed that the odds of having high TG in the alcohol-drinking group in the last 12 months was 1.53 times higher than in the non-drinking group.

Weitzman et al. showed environmental exposure to tobacco smoking and active tobacco smoking were independently associated with the MetS among 2273 subjects aged 12 to 19 years in the United States ^39^. A Meta-analysis study showed hookah smoking increased the risk of obesity among all age groups, regardless of gender. Also, this association remained significant after a correction for several confounders ^40^. Therefore, hookah smoking seems to be associated with a higher risk of obesity. The results of our study also confirmed this result and showed that continuous use of hookah increases waist circumference which ultimately causes MetS.

The main strengths of the present study were population-based study and a large sample size, which minimizes the occurrence of random error and reinforces the reliability of our analyses. Besides, anthropometric characteristics were measured by trained personnel according to the PERSIAN cohort protocols, and data on the lipid profile of all participants were measured by one type of device and a single laboratory.

Our study has several important limitations. First, this study was a cross-sectional study so, we could not prove any cause-effect association between personal habits and MetS. Therefore, the associations between MetS and personal habits require validation in future large-scale longitudinal and other cohort studies. Second, since MetS can be influenced by the diet, dietary intake was not available in our study population. Another important limitation was that alcohol and opium consumption, tobacco use, and cigarette smoking were self-reported, therefore, may cause measurement bias. However, the amount of this self-reporting bias depends on sex, age, type of substances, geographical area, and the understudy population. It can be a result of the differences in social and cultural beliefs of various individuals and regions. Fortunately, we believe that the validity of the data on opium use in our population is relatively high. The our previous study about the validity of self-reported substance use among the RYCS showed that the self-reported validity of opium use during the past year is relatively good and the level of agreement increased with longer recall periods ^41^.

## Conclusion

The prevalence of MetS was 7.67%, 7.14%, and 10.13% based on NCEP-ATPIII, IDF Iranian, and International IDF criteria respectively. The odds of MetS and high TG were greater in the alcohol-drinking group in the last 12 months compared with the non-drinking group. The odds of high WC in the last 12 months, were greater in the tobacco-smoking group compared with the non-smoking group. However, more longitudinal studies are needed to verify the associations observed in the current study.

### Supplementary Information

Supplementary Information 1.

Supplementary Table S1.

Supplementary Table S2.

## Data Availability

The data is not available publicly. However, upon a reasonable request, the data can be obtained from the corresponding author (Parvin Khalili).
