# Supplementary material for: Metabolic syndrome: a population-based study of prevalence and risk factors
Source: Sci Rep. 2024 Feb 17;14:3987. doi: 10.1038/s41598-024-54367-4 (PMC10874377; doi:10.1038/s41598-024-54367-4)
Supplement: Supplementary file 1 — Supplementary Information 1. [file 41598_2024_54367_MOESM1_ESM.docx]

The questionnaires (physical activity, and personal habits) used in this study were part of the PERSIAN cohort study questionnaires including:

**The physical activity:**

" During the past week, how many days did you exercise or do moderate to vigorous physical activity for at least 10 minutes?"

" During the past week, on days you did moderate to vigorous physical activity for at least 10 minutes, on average how many minutes a day did it take?".

“Moderate to vigorous physical activity” is defined as activities, which can lead to increased breathing rate.

**Personal habits:**

**Opium consumption:**

Opium consumption life time: Have you consumed opium or its derivatives in your lifetime?

Opium consumption in the last 12 m: Have you consumed opium or its derivatives in the last 12 months?

**Cigarette smoking**

Cigarette smoking life time: Have you smoked at least one whole cigarette in your lifetime?

Cigarette smoking life time daily: Have you smoked cigarette every day for 1 month or more in your lifetime?

Cigarette smoking in the last 12m: Have you smoked at least one whole cigarette in the last 12 months?

Cigarette smoking in the last 12 m daily: Have you smoked cigarette every day for 1 month or more in the last 12 months?

**Tobacco consumption**

Tobacco consumption life time: Have you used hookah or pipe in your lifetime?

Tobacco consumption life time daily: Have you used hookah or pipe every day for 1 month or more in your lifetime?

Tobacco consumption in the last 12m: Have you used hookah or pipe in the last 12 months?

Tobacco consumption in the last 12 m daily: Have you used hookah or pipe every day for 1 month or more in the last 12 months?

**Alcohol drinking:**

Alcohol drinking life time: Have you consumed alcoholic drinks in your lifetime?

Alcohol drinking in the last 12 m: Have you consumed alcoholic drinks in last 12 months?
