# Supplementary material for: Metabolic syndrome: a population-based study of prevalence and risk factors
Source: Sci Rep. 2024 Feb 17;14:3987. doi: 10.1038/s41598-024-54367-4 (PMC10874377; doi:10.1038/s41598-024-54367-4)
Supplement: Supplementary file 2 — Supplementary Table S1. [file 41598_2024_54367_MOESM2_ESM.docx]

| Table S1. Association of opium consumption, cigarette and tobacco smoking and alcohol drinking with Mets according to IDF international, IDF Iranian, and NCEP-ATP III criteria based on age group. | | | | | | | |
| --- | --- | --- | --- | --- | --- | --- | --- |
|  | | **MetS (IDF international)** | | **MetS (IDF Iranian)** | | **MetS (NCEP**-**ATP III)** | |
|  |  | Adjusted (15-35years, n=2843) | Adjusted (19-35years, n=2352) | Adjusted (15-35years, n=2843) | Adjusted (19-35years, n=2352) | Adjusted (15-35years, n=2843) | Adjusted (19-35years, n=2352) |
| **Opium consumption in the last 12 months** | Yes | 0.98(0.63-1.52) | 0.98(0.63-1.52) | 0.92(0.56-1.49) | 0.92(0.56-1.49) | 1.01(0.61-1.67) | 1.01(0.61-1.68) |
|  | No | 1 | 1 | 1 | 1 | 1 | 1 |
| **Opium in life time** | Yes | 1.20(0.84-1.71) | 1.17(0.82-1.67) | 1.06(0.72-1.57) | 1.02(0.68-1.52) | 1.15(0.76-1.73) | 1.11(0.73-1.68) |
|  | No | 1 | 1 | 1 | 1 | 1 | 1 |
| **Opium abuse** | Yes | 1.18(0.51-2.74) | 1.15(0.50-2.67) | 0.96(0.39-2.35) | 0.93(0.38-2.30) | 0.67(0.20-2.25) | 0.66(0.20-2.20) |
|  | No | 1 | 1 | 1 | 1 | 1 | 1 |
| **Opium dependent** | Yes | 1.27(0.60-2.69) | 1.25(0.59-2.65) | 1.08(0.49-2.39) | 1.07(0.48-2.36) | 0.75(0.26-2.16) | 0.74(0.26-2.14) |
|  | No | 1 | 1 | 1 | 1 | 1 | 1 |
| **Cigarette smoking in the last 12 months** | Yes | 1.19(0.83-1.70) | 1.22(0.84-1.76) | 1.26(0.85-1.85) | 1.30(0.87-1.92) | 1.38(0.90-2.10) | 1.43(0.93-2.20) |
|  | No | 1 | 1 | 1 | 1 | 1 | 1 |
| **Cigarette smoking in the last 12 months daily** | Yes | 1.39(0.08-2.41) | 1.39(0.80-2.41) | 1.45(0.83-2.53) | 1.45-(0.83-2.53) | 1.41(0.73-2.72) | 1.42(0.73-2.74) |
|  | No | 1 | 1 | 1 | 1 | 1 | 1 |
| **Cigarette smoking in lifetime** | Yes | 1.00(0.72-1.39) | 1.00(0.71-1.41) | 0.95(0.66-1.38) | 0.95(0.65-1.39) | 1.05(0.72-1.55) | 1.10(0.74-1.63) |
|  | No | 1 | 1 | 1 | 1 | 1 | 1 |
| **Cigarette smoking in lifetime daily** | Yes | 1.25(0.77-2.03) | 1.24(0.76-2.02) | 1.23(0.75-2.03) | 1.22(0.74-2.02) | 1.19(0.66-2.14) | 1.19(0.66-2.14) |
|  | No | 1 |  | 1 | 1 | 1 | 1 |
| **Alcohol drinking in the last 12 months** | Yes | **1.51(1.02-2.21)** | **1.58(1.06-2.35)** | **1.66(1.11-2.48)** | **1.77(1.17-2.68)** | 1.26(0.78-2.03) | 1.31(0.81-2.13) |
|  | No | 1 |  | 1 | 1 | 1 | 1 |
| **Alcohol drinking lifetime** | Yes | 1.27(0.87-1.84) | 1.28(0.86-1.90) | 1.23(0.82-1.83) | 1.24(0.81-1.89) | 0.96(0.61-1.52) | 0.94(0.58-1.50) |
|  | No | 1 |  | 1 | 1 | 1 | 1 |
| **Alcohol abuse** | Yes | 1.41(0.78-2.55) | 1.40(0.77-2.56) | 1.35(0.73-2.49) | 1.36(0.73-2.53) | 1.27(0.62-2.73) | 1.30(0.61-2.75) |
|  | No | 1 | 1 | 1 | 1 | 1 | 1 |
| **Alcohol dependent** | Yes | 0.96(0.42-2.17) | 0.97(0.42-2.20) | 0.99(0.44-2.26) | 1.01(0.44-2.33) | 1.08(0.42-2.82) | 1.10(0.42-2.88) |
|  | No | 1 | 1 | 1 | 1 | 1 | 1 |
| **Tobacco smoking in the last 12 months** | Yes | 1.14(0.86-1.50) | 1.19(0.89-1.59) | 1.16(0.84-1.61) | 1.21(0.87-1.70) | 1.27(0.92-1.75) | 1.30(0.93-1.81) |
|  | No | 1 | 1 | 1 | 1 | 1 | 1 |
| **Tobacco smoking in the last 12 months daily** | Yes | 1.24(0.76-2.03) | 1.24(0.75-2.05) | 1.40(0.84-2.33) | 1.44(0.86-2.41) | 1.47(0.84-2.57) | 1.48(0.84-2.61) |
|  | No | 1 | 1 | 1 | 1 | 1 | 1 |
| **Tobacco smoking in lifetime** | Yes | 0.95(0.72-1.26) | 0.97(0.73-1.31) | 0.87(0.62-1.21) | 0.89(0.62-1.27) | 0.90(0.65-1.24) | 0.90(0.65-1.26) |
|  | No | 1 | 1 | 1 | 1 | 1 | 1 |
| **Tobacco smoking in lifetime daily** | Yes | 1.09(0.72-1.67) | 1.07(0.70-1.65) | 1.22(0.79-1.88) | 1.22(0.78-1.90) | 1.16(0.70-1.91) | 1.15(0.69-1.91) |
|  | No | 1 | 1 | 1 | 1 | 1 | 1 |
| Adjusted model was adjusted for confounding variables age (continuous variable), gender (male/female), and education years (continuous variable).  Abbreviations: MetS: Metabolic syndrome. | | | | | | | |
