# Supplementary material for: Metabolic syndrome: a population-based study of prevalence and risk factors
Source: Sci Rep. 2024 Feb 17;14:3987. doi: 10.1038/s41598-024-54367-4 (PMC10874377; doi:10.1038/s41598-024-54367-4)
Supplement: Supplementary file 3 — Supplementary Table S2. [file 41598_2024_54367_MOESM3_ESM.docx]

| Table S2. Sensitivity analysis by multiple imputation strategy for association of opium consumption, cigarette and tobacco smoking and alcohol drinking with Mets according to NCEP-ATP III criteria. | | |
| --- | --- | --- |
| **Personal habits** | | **MetS**  **Adjusted OR (95% CI)** |
| **Opium consumption in the last 12 months** | Yes | 1.03(0.62-1.70) |
|  | No | 1 |
| **Opium in life time** | Yes | 1.17(0.77-1.76) |
|  | No | 1 |
| **Opium abuse** | Yes | 0.60(0.18-2.00) |
|  | No | 1 |
| **Opium dependent** | Yes | 0.71(0.25-2.03) |
|  | No | 1 |
| **Cigarette smoking in the last 12 months** | Yes | 1.36(0.89-2.10) |
|  | No | 1 |
| **Cigarette smoking in the last 12 months daily** | Yes | 1.43(0.74-2.75) |
|  | No | 1 |
| **Cigarette smoking in lifetime** | Yes | 1.04(0.71-1.53) |
|  | No | 1 |
| **Cigarette smoking in lifetime daily** | Yes | 1.17(0.65-2.12) |
|  | No | 1 |
| **Alcohol drinking in the last 12 months** | Yes | 1.25(0.78-2.01) |
|  | No | 1 |
| **Alcohol drinking lifetime** | Yes | 0.98(0.63-1.54) |
|  | No | 1 |
| **Alcohol abuse** | Yes | 1.27(0.61-2.64) |
|  | No | 1 |
| **Alcohol dependent** | Yes | 1.02(0.39-2.68) |
|  | No | 1 |
| **Tobacco smoking in the last 12 months** | Yes | 1.23(0.90-1.68) |
|  | No | 1 |
| **Tobacco smoking in the last 12 months daily** | Yes | 1.46(0.84-2.53) |
|  | No | 1 |
| **Tobacco smoking in lifetime** | Yes | 0.89(0.65-1.23) |
|  | No | 1 |
| **Tobacco smoking in lifetime daily** | Yes | 1.15(0.70-1.88) |
|  | No | 1 |
